# Neglected Pessary in Patient With Pelvic Organ Prolapse: A Case Report 

**Published:** 2019-09

**Authors:** Zinat Ghanbari, Maryam Deldar-Pesikhani, Tahereh Eftekhar, Leila Pourali, Atiyeh Vatanchi, Soudabeh Darvish, Elnaz Ayati

**Affiliations:** 1 Department of Obstetrics and Gynecology, School of Medicine, Tehran University of Medical Sciences, Tehran, Iran; 2 Department of Obstetrics and Gynecology, Faculty of Medicine, Mashhad University of Medical Sciences, Mashhad, Iran; 3 Department of Obstetrics and Gynecology, Faculty of Medicine, Shahid Beheshti University of Medical Sciences, Tehran, Iran

**Keywords:** Pessary, Pelvic Organ Prolapse, Conservative Treatment

## Abstract

**Objective:** To report the neglected pessary in a patient with pelvic organ prolapse. Pelvic organ prolapse (POP) is one of the most important medical challenges in women especially elderly. One of the conservative treatments of symptomatic POP is pessary placement.

**Case report:** A 84-year-old woman, para 10 was referred to female pelvic floor clinic of an academic hospital for vaginal bleeding and neglected vaginal pessary. Vaginal examination in the pelvic floor clinic revealed an entrapped ring pessary in severely atrophic vaginal mucosa with purulent discharge.

**Conclusion:** Although pessary is the first choice and one of the best conservative treatment for pelvic organ prolapse, it shouldn’t be used for poor cooperative patient who cannot comply with regular follow-up visits which may cause harmful complications.

## Introduction

Pelvic organ prolapse (POP) is one of the most important medical challenges in women especially elderly; considering it’s negative effect on quality of life and bothersome symptoms like vaginal pressure, dyspareunia, incomplete bladder or rectal emptying (1). One of the conservative treatments of symptomatic POP is pessary placement. Indeed pessary trial is a low risk option for women experiencing symptomatic POP and should be considered and offered routinely (2). The most important contraindication of pessary is noncompliance with follow-up as this neglected pessary can result in severe complications like vesicovaginal or rectovaginal fistula and hydrouretero nephrosis (3). To the extent of our knowledge, this is the first case report of neglected pessary in Iran.

## Case report

A 84-year-old Iranian woman, para 10 was referred to female pelvic floor clinic of Imam Khomeini hospital, Tehran University of Medical Sciences, Tehran, Iran for vaginal bleeding and neglected vaginal pessary. Ten years ago, a ring pessary was administered to control the vaginal vault prolapse symptoms. Despite the medical team recommendation, she didn’t have regular follow-up visits since 9 years ago. When vaginal bleeding occurred she referred to her physician, vaginal examination showed the pessary entrapped to the vaginal mucosa and she referred to an academic hospital of Tehran University of Medical Sciences. 

**Figure 1:**
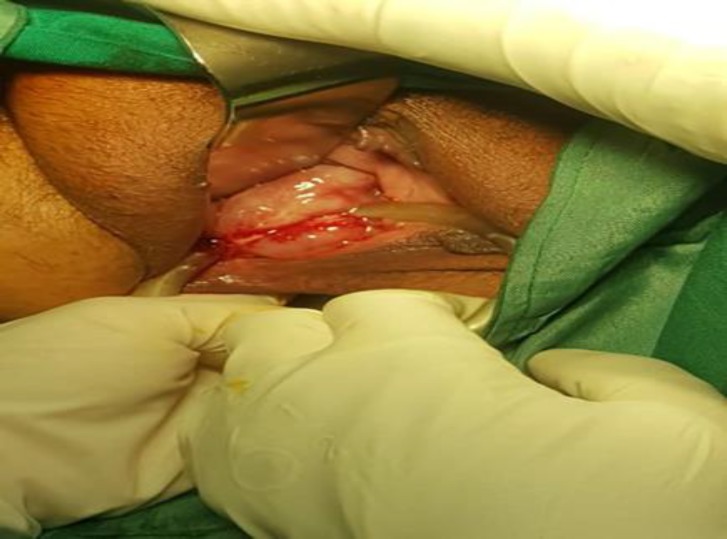
Entrapment of ring pessary in vaginal mucosa

Vaginal examination in the pelvic floor clinic revealed an entrapped ring pessary in severely atrophic vaginal mucosa with purulent discharge. There was some fibrotic bundle between vaginal mucosa and pessary which entrapped the pessary in the vaginal mucosa. Rectal examination was normal and there was no obvious rectovaginal or vesicovaginal fistula (Figure 1).

**Figure 2:**
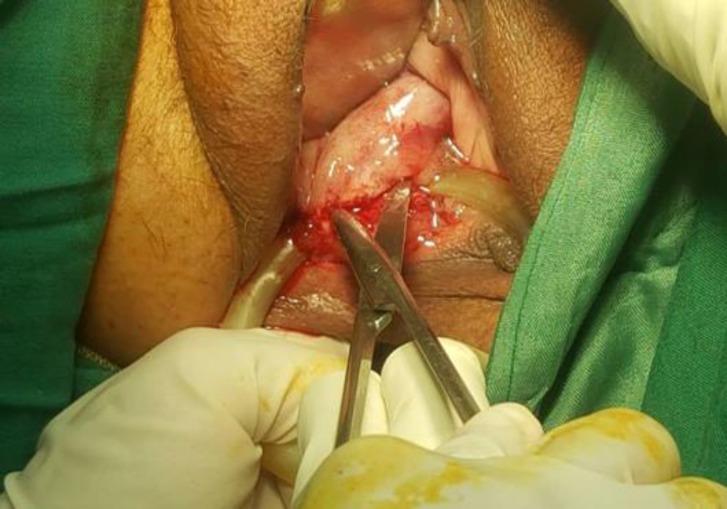
Fibrotic bundles between vaginal mucosa and pessary excised with Metz scissors

After prescribing vaginal estrogen and antibiotic (oral metronidazole 500 mg twice a day) for 10 days, under spinal analgesia impacted pessary was dissected from fibrotic vaginal bundles (Figure 2).

With regard to the persistent urinary symptoms (frequency and urge urinary incontinence), cystoscopy was performed which revealed hypervascularity, trabeculation and diverticulum in the bladder base. This finding might be cause of bladder overactivity, so we started medical treatment to decrease the urinary symptoms. She was advised to use vaginal estrogen 3 times a week for improvement of vaginal atrophy. Informed consent was obtained from patient to publish the case presentation and related figures.

## Discussion

Although pessary is a safe alternative treatment instead of surgery, informing and educating patients and their care givers for regular follow-up visits are the important issue in this field. Patient's refuse for regular follow-up is the most important contraindication for pessary usage. According to optimal follow-up recommendations noted in gynecology text books after the initial fitting, the patient should return in 1 to 2 weeks and then at 4-6 weeks (1). After this initial follow-up, depending on the patient’s ability to insert and remove the pessary, she is advised for regular follow-up visits every 1-3 months for dependent and every 6-12 months for independent users (2). In each visit, the pessary should be removed and cleaned with soap and water and the vagina should be inspected for erosion, ulceration or abnormal discharge. Vaginal estrogen should be administered for better vaginal lubrication and lowered the risk of erosion and ulseration (1, 4). The current case refused to follow-up visit for about 9 years. 

Manivasakan reported a 55 years old woman with encapsulated vaginal pessary. She had no follow-up visit since the first insertion time about 3 years ago. Just like the current patient. Fortunately their case had no rectal or urinary system complications. Her chief complain was mass descending per vagina which was not the same as our patient (5).

Another case report presented a 72 years old female who using vaginal pessary since 12 years ago without regular follow-up. She complained of foul smelling discharge for one year. Like the current case, her ring pessary buried in atrophic vaginal mucosa which needed excision under general anesthesia for pessary removal (6). In some other reports also more dangerous complications happened in the case of poor pessary follow-up visits. Arias et al. reported a 89 years old woman with neglected pessary which presented with vesicovaginal fistula. In contrast to our case, her pessary was gelhorn type that is an space filling pessary (7). 

Thuraya et al. reported a 77 years old woman which her neglected pessary perforated the small bowel. Like the current case, her pessary was ring type (8). In addition to vesicovaginal and rectovaginal fistula, neglected pessary can cause hydrouretero nephrosis which may lead to harmful consequences. (9).If abnormal uterine bleeding did not occurred in current patient ,maybe she did not refer to physician and more pessary penetration caused much harmful consequences.

## Conclusion

Although pessary is the first choice and one of the best conservative treatment for pelvic organ prolapse, it shouldn’t be used for poor cooperative patient who cannot comply with regular follow-up visits which may cause harmful complications.
